# Livelihood strategies, capital assets, and food security in rural Southwest Ethiopia

**DOI:** 10.1007/s12571-018-00883-x

**Published:** 2019-01-24

**Authors:** Aisa O. Manlosa, Jan Hanspach, Jannik Schultner, Ine Dorresteijn, Joern Fischer

**Affiliations:** 1 Faculty of Sustainability, Leuphana University Lueneburg, Lueneburg, Germany; 2 Copernicus Institute of Sustainable Development, Utrecht University, Utrecht, Netherlands

**Keywords:** Livelihood strategies, Food policies, Smallholder farming, Agriculture, Diversification, Ethiopia

## Abstract

Households combine capital assets in a process involving human agency and resourcefulness to construct livelihood strategies and generate well-being outcomes. Here, we (1) characterized types of livelihood strategies; (2) determined how different capital assets are associated with different livelihood strategies; and (3) determined how livelihood strategies differed in food security outcomes. We conducted a survey in southwestern Ethiopia and used principal component and cluster analyses. Five types of livelihood strategies, which differed mainly in food and cash crops comprising the strategy, were identified. These were, in order of decreasing food security: ‘three food crops, coffee and khat’, *n* = 68; ‘three food crops and khat’, *n* = 59; ‘two food crops, coffee and khat’, *n* = 78; ‘two food crops and khat’, *n* = 88; and ‘one food crop, coffee and khat’, *n* = 44. The livelihood strategy ‘three food crops, coffee and khat’ was associated with a wide range of capital assets, particularly having larger aggregate farm field size and learning from other farmers. A generalized linear model showed that livelihood strategies were significantly associated with food security outcomes. Particularly, a high number of food crops in a strategy was linked with relatively high food security. In this context, diversified livelihood strategies primarily through having a mix of food crops for subsistence, in combination with cash crops for income, are important for food security. This suggests a need to rethink dominant policy narratives, which have a narrow focus on increasing productivity and commercialization as the primary pathway to food security.

**Electronic supplementary material:**

The online version of this article (10.1007/s12571-018-00883-x) contains supplementary material, which is available to authorized users.

## Introduction

Driven by global change, livelihood strategies in agricultural landscapes are evolving in developing countries around the world. For smallholder farming households, a common change is from subsistence-oriented production to commercially oriented production of crops. Such a shift is actively encouraged by some governments (see e.g. Gebrehiwot et al. 2016; Vongvisouk et al. 2014) on the grounds that it will improve food security through economic growth. However, outcomes of such a change have been mixed so that the ways in which different livelihood strategies influence household food security in different settings is less clear (Lang and Barling 2012). Understanding how livelihood strategies, particularly different combinations of food crops and cash crops, influence the food security of smallholder farming households is important for identifying and supporting sustainable development trajectories of traditionally subsistence-oriented or semi-subsistent agricultural landscapes.

For smallholder farming households, two plausible pathways of crop production have been advocated to increase food security, namely: (1) cash crop production (e.g. Achterbosch et al. 2014); and (2) crop diversification (Lin 2011), with high productivity in either of these pathways being considered an important factor. Maxwell and Fernando (1989) defined cash crops as all marketed surplus, non-staple agriculture, non-food agriculture, and export agriculture. Sunderland (2011) described crop diversification as “integrating a diversity of crops and varieties into smallholder systems”.

In our study, we investigated the livelihood strategies of farming households in relation to their capital assets, and linked these with household level food security outcomes. We considered different combinations of livelihood activities, which, in the context studied, primarily consisted of food crops and cash crops. We focused on Ethiopia where, in 2015, about 81% of the population lived in rural areas and mainly relied on agriculture for their livelihoods (World Bank 2016). We selected southwest Ethiopia, an area with high biodiversity, large tracts of Afromontane forests (Hylander et al. 2013), and home to the wild gene pool of Arabica coffee (*Coffea arabica*), which generates the largest foreign exchange for the country (FAO 2016). Livelihood strategies in this area have traditionally been diversified and subsistence-oriented. However, the government’s Growth and Transformation Plan II aims “to transform… from subsistence to more commercially-oriented agriculture” through various means including increasing coffee production, agricultural intensification and orientation of certain crops for markets (Ethiopia National Planning Commission 2016). Within government circles, this trajectory from subsistence to commercial orientation is perceived as promising potential benefits for food security. Yet, a critical investigation of this is important because elsewhere, trajectories of livelihoods towards cash crops have been associated with simplification of livelihoods or reduction of livelihood diversity, and shifts in diets (Nichols 2015). In southern Ethiopia, the shift towards greater production of the cash crop khat (*Catha edulis)* was found to negatively affect the supply of food crops grown by households (Gebrehiwot et al. 2016).

Against this context of changing livelihoods and government incentives, our objectives were to (1) develop an empirically grounded characterization of existing livelihood strategies in the study area; (2) determine how different types of capital asset are associated with different livelihood strategies; and (3) examine how the identified livelihood strategies differ in terms of food security outcomes. Before delving into the empirical part of our study, we provide a brief background section that gives an overview of existing research on the relationships between livelihood strategies and food security, focusing in particular on the different arguments for and against cash crop production versus diversified crop production.

## Background on the relationships between livelihoods and food security

Determining how food security can be achieved has been a long-standing subject of scholarly and policy debates. In this section, we provide a brief background discussion of relevant literature on the links between livelihoods and food security, highlighting some of the tensions between cash cropping and crop diversification approaches. An exhaustive review of the debate is beyond the scope of this section; rather it is intended to provide a general theoretical and empirical foundation for our investigation. We first outline developments in livelihoods research and then transition into the more specific debate on how different kinds of livelihoods relate to food security.

Sustainable livelihoods thinking has contributed rich understandings of the ways individuals, households, and social groups in different contexts exercise agency and use their capital assets to produce outcomes necessary for sustenance and well-being (de Haan and Zoomers 2006; Levine 2014). The seminal work by Chambers and colleagues (Chambers 1987; Chambers and Ghildyal 1985; Chambers and Conway 1992) emphasized placing people at the center of scientific inquiry into poverty, food security, and environmental degradation and gave rise to livelihoods thinking. Subsequently, certain principles of livelihoods thinking were operationalized through the formulation of the Sustainable Livelihoods Framework (Carney 1999; Scoones 1998), or in short, the “livelihoods approach”. The livelihoods approach has been widely used for systematically analyzing livelihoods and their relationships with well-being outcomes, both in rural and urban areas. Often, the critical question is how different livelihood strategies generate different outcomes for individuals, households, or groups in terms of incomes, nutrition, caloric intake, or other well-being measures (e.g. Frison et al. 2011; Martin et al. 2013). In rural areas particularly, the multi-faceted nature of agricultural livelihoods, the dynamism of contexts, temporality, and the element of human agency responding to and acting on accessible capital assets make it challenging to generalize which livelihood strategies generate the best outcomes for human well-being. Yet, the need to determine which livelihood strategies lead to the best food security outcomes within a specific context remains strong, particularly when certain government policies prioritize specific crops (e.g. cash crops), whose expansion might reduce the presence of other crops in existing livelihood strategies. A better understanding of the food security outcomes associated with different livelihood strategies is particularly important in semi-subsistence landscapes. Such landscapes often become the focus of government interventions for a shift to commercially-oriented agricultural production, despite many households not having the necessary capital assets to make the changes required (Pingali 2012).

Improving food security through the cash crop pathway is premised on the production and marketing of cash crops (or of commercially-oriented food crops) to generate financial income that farming households can use not only to purchase food, but also to accumulate capital assets necessary for further improving their livelihoods (Govereh and Jayne 2003). This pathway ultimately aims to address poverty, which is an important cause of food insecurity (Smith et al. 2000). Cotton production in Gokwe North District, Zimbabwe (Govereh and Jayne 2003) and palm oil production in Indonesia (Sayer et al. 2012) exemplify the potential economic benefits (and indirectly food security benefits) resulting from intensive engagement in cash crop production. However, consequences are not always positive particularly for the poor; and diverging outcomes have been observed for different community groups. For example, the cash crop sugarcane was found to have a positive effect on food security in Ethiopia, but cotton production in Ghana resulted in lower food security among growers (Lam et al. 2017). In Sulawesi, Indonesia, Belsky and Siebert (2003) found that food self-sufficiency would likely decline with conversion of food-crop focused swidden fields to cocoa farms. In northern Vietnam, intensified and commercialized agriculture linked with cash crops also suggested the emergence of “new food insecurities and vulnerabilities” (Bonnin and Turner 2012). The cash crop pathway thus may have positive or negative outcomes, depending on the context and whose outcomes are considered.

The crop diversification pathway may benefit food and nutrition security primarily by enabling households to have direct access to staples and other types of food crops (Jones et al. 2014; Powell et al. 2015). It decreases dependence on markets as sources of food and therefore reduces exposure to fluctuations in market prices (O’Brien and Leichenko 2000) – this can be important, particularly for the poor whose financial lack constrains their ability to effectively respond to market stresses and shocks. Food crop diversification also enables households to spread risks over different crop types so that failure in one does not lead to the collapse of the entire livelihood strategy (Ellis 2000). In the Bolivian Andes, production of diverse food crops for subsistence was found to be a plausible approach for improving household and children’s diets (Jones 2014). In Kenya, agricultural diversity consisting mostly of food crops was positively related to nutrient adequacy ratios (M’Kaibi et al. 2015). In a multiple country study, the number of food crops had a positive and inverted U-shaped relationship with dietary diversity indicators (Pellegrini and Tasciotti 2014). That is, dietary diversity increased with crop diversity up to a point and then began to decrease. However, in most studies it remains unclear whether the positive effects of crop diversification resulted directly from consumption of the food crops, or through selling them.

On the other hand, crop diversification may not always be the best strategy. Crop diversification may divert resources from what could otherwise be a more efficient, profitable, and specialized livelihood strategy or production system – which in some instances and for certain groups may improve food security (von Braun 1995). Subsistence-based diversification strategies also do not primarily facilitate income generation. This is important because higher income from agricultural production has been found to be associated with improved food security (e.g. Salazar et al. 2015). Similarly, Sibhatu and Qaim (2018) found that subsistence production contributed less to dietary diversity than cash income. Such mixed outcomes across different contexts suggest that pathways towards food security need to be grounded in a contextualized understanding of existing livelihood strategies.

The construction of livelihood strategies can be seen as the outcome of an actively negotiated process where households consider available capital assets, achievable household goals, and options for realizing these goals within the limits of capital assets (Rakodi 1999). Analyzing existing livelihood strategies and outcomes in a specific context is primal because context shapes the opportunity structures within which livelihoods are constructed (Bebbington 1999). For example, how well an area is connected to markets, and the extent to which transportation facilities are accessible, may influence the livelihood strategies in an area (Acheampong et al. 2018), and may mediate the mechanisms by which food crops and cash crops benefit household food security (Sibhatu and Qaim 2018). Moreover, the ability of households to engage in a type of livelihood strategy is influenced by the types of capital assets they have access to (Scoones 1998; Rakodi 1999). We hypothesized that differentiated access to capital assets such as land, livestock and social capital enable or constrain types of livelihood strategies.

## Material and methods

### Study area and field sampling

We studied six *kebeles* (smallest administrative unit in Ethiopia) situated in three *woredas*, or districts, in Jimma Zone, Oromia Region, Ethiopia. *Kebeles* were selected along an altitude and forest cover gradient to capture a variety of livelihood strategies (Online Resource 1). The highlands of southwest Ethiopia receive an average of 2275 mm of annual rainfall, with a rainy period from February to November (Kidanewold et al. 2014). By international standards, food security is low (Ethiopia CSA and WFP 2014) particularly during the lean season from June to August every year. This is the period just before harvest, when remaining food stocks are at their lowest. The number of households in the *kebeles* ranged from 322 to 1222. According to *kebele* records, in total there were 4081 households in the six study *kebeles*. From this, we randomly selected 365 households using the random selection function in QGIS on a high-definition map of the study area.

### Survey tool and concepts used

We used a survey questionnaire for data collection. This was implemented with the assistance of two trained enumerators. The survey tool was translated into the local language *Aafan Oromo* and back-translated to English to ensure that the integrity of the original meaning was maintained. It was pre-tested in a pilot study in August 2015, and revised before the data collection period, which ran from November 2015 to January 2016. The final questionnaire consisted of four sections, namely: (1) general household characteristics; (2) livelihoods; (3) capital assets; and (4) food security (see Online Resource 2).

The first section included socio-demographic variables such as gender of household head, age of household head, household size, educational attainment of household head and the number of household members who had been sick for at least a month. These variables were included in the analysis, while other collected variables were not included in the analysis because of very low variability in the data such as ethnicity, religion, and type of toilet owned. The second and third sections were guided by the Sustainable Livelihoods Framework. We defined livelihoods as being comprised of the *strategies* and *assets* required to make a living (Scoones 1998). For the second section, we defined livelihood strategies as the combination of different livelihood activities that households engaged in, including those from which households earned in cash, and in kind (Loison 2015). We asked about all types of livelihood activities to determine the composition of livelihood strategies. Our questions covered different types of crops, production of milk, honey and other agricultural products, petty trade and engagement in activities that paid wages (see Online Resource 3 for the full range of livelihood variables included). Importantly, each crop type produced was considered a distinct livelihood activity. For the third section, we considered *capital assets* as the building blocks from which households constructed livelihood strategies. Here, questions related to various capital asset variables belonging to one of five *capital asset* types (i.e. economic, human, natural, physical, and social). Some examples under economic capital assets were access to credit and having a coffee plot. For human capital, we included questions on health and access to information or knowledge through formal or informal channels (Table 1). The fourth section on food security was a modified version of the Household Food Insecurity Access Scale (HFIAS) (Coates et al. 2007; Maxwell et al. 2013). Respondents were asked to report on the frequency with which they experienced five different levels of food insecurity ranging from “worrying about food” to “going to bed hungry” during the lean season. The frequency of each experience was scored: zero (not experienced), one (rarely, about once or twice a month), two (sometimes, about three to ten times a month), or three (often, estimated more than ten times a month). The scores enabled us to derive a total HFIAS score ranging from 0 to 15 for each household, with smaller values indicating high food security and higher values indicating low food security. Between two months and five months had passed since the end of the lean season from the first household to the last household surveyed. This recall period was longer than used in most other studies. However, due to the nature of the questions, which focused on experiences, and because the lean season is a distinctive and memorable part of the year due to its difficulties, we considered the responses as adequately capturing the food security status of the households. To statistically confirm this, we designed our model to detect effects from temporal proximity of each survey date to the lean period, by incorporating survey date as a variable in the model used (see below). Modified versions of the HFIAS have been found to be robust tools for assessessing food security in other parts of Ethiopia (Gebreyesus et al. 2015). The survey was implemented such that the first half of the sample in each *kebele* was completed during the first half of the field work. We then returned to every *kebele* to complete the survey in the second half of the field work. In addition to the survey, we also took field notes to record qualitative observations concerning the broader context such as physical infrastructure, market access, and livelihood problems, and gained insights from informal conversations with local residents.

**Table 1:** List of capital asset variables included in analysis and how each variable was measured

Type of capital asset	Variable	Measurement
Economic	Access to credit	0 – No, 1 – Yes
	Ownership of coffee plot	0 – No, 1 – Yes
	Ownership of khat plot	0 – No, 1 – Yes
Human	Learning farming-related information from development agents	0 – No Yes, Frequency 1 – Rarely 2 – Seldom 3 – Often
	Learning farming-related information from other farmers,	0 – No Yes, Frequency 1 – Rarely 2 – Seldom 3 – Often
	Family farm labor	Number of family members that help in the farm
	Access to information about new technology and market prices	0 – No Yes, Frequency 1 – Rarely 2 – Seldom 3 – Often
	Highest educational attainment of household head	0 – No education 1 – Adult education or special education 2 – Grades 1 to 6 3 – Grades 7 to 12 4 – Grades 13 and above
	Health using as proxy presence or absence of household members who got sick continuously for more than a month in the last one year	1 – Yes 0 – No
Natural	Access to surrounding natural resources such as forests and water	0 – No, 1 – Yes
	Perception on environmental change in the immediate landscape, whether positive or negative	0 – No change or worsening 1 – Improving
	Perception on soil fertility	0 – Bad 1 – Medium 2 – Good
	Access to trees for the production of honey	0 – No, 1 – Yes
	Access to eucalyptus	0 – No, 1 – Yes
	Size of farm fields	Total size in hectares
	Size of home garden	Total size in hectares
	Land rights (whether having a land certificate or not)	0 – No, 1 – Yes
Physical	Length of travel time to get from house to market	Minutes
	Livestock and poultry owned	Number of livestock and poultry
	Mobile phone owned	Number of mobile phones
	Farm tools owned	Number of farm tools
Social	Membership to farming organization	0 – No, 1 – Yes
	Presence or absence of individuals or organizations to turn to for help with livelihood problems	0 – No, 1 – Yes
	Presence or absence of individuals or organizations to turn to for help with shortage in food or cash income	0 – No, 1 – Yes
	Ability to speak out regarding management of nearby natural resources	0 – No, 1 – Yes
	Sharing or borrowing of livestock	Number of livestock used (i. e. for farming) which was either borrowed or within a livestock-sharing arrangement
	Sharecropping	Number of crops that were produced through sharecropping arrangements

### Data analysis

We processed the data in R (R Development Core Team 2008). As a first step, we explored the distribution and variability of data. Variables with very low variability across the households were excluded from the analysis. For the variables that were selected for inclusion in the analysis, we identified cells with missing data and applied an imputation process called multiple imputation chained equations through the ‘mice’ package in R (Van Buuren and Groothuis-Oudshoorn 2011). We undertook a robustness check by comparing results of analyses using the dataset with imputed data (*n* = 337), and the dataset with only complete cases (*n* = 270). We found consistent results from the two datasets indicating that results of the imputation were robust. A total of 337 questionnaires were used for the final analysis. We then visually inspected distributions of the continuous data and log-transformed skewed variables to meet requirements of normality for multivariate analyses.

Qualitative data from field notes were used to provide a descriptive background of the local context. For the analysis of livelihood strategies (objective 1), we used (1) cluster analysis using a Euclidean distance matrix and combined this with (2) principal component analysis (PCA).^1^ We applied Ward hierarchical clustering because this yielded a clear group structure and better interpretability of results than other clustering methods. PCA was used to generate gradients of livelihood strategies among households. Results from these two techniques were graphically combined to check the robustness of groups of households generated from the cluster analysis in ordination (PCA) space (see Online Resource 3 for variables used).

Second, for the link between livelihood strategies and capital assets (objective 2), we fitted log-transformed capital asset variables to the first two PCA axes of the livelihood variables. Specifically, using the ‘envfit’ function in R (Oksanen et al. 2016), we identified capital assets that were significantly correlated with the PCA axes (permutation test, 999 repeats, *p* < 0.01). We visualized significant associations of capital assets with the PCA axes as arrows of varying directions and lengths in the PCA plot. This enabled us to interpret the association of different types of capital assets with different livelihood strategies. As a further step, using multinomial logistic regression, we tested for relationships between livelihood strategies as a categorical response variable against capital asset variables with significant associations from the envfit analysis (multinom function from the nnet package) (Venables and Ripley 2002). Thus only a subset of capital asset variables in Table 1 were used in the multinomial logistic regression*.* We emphasize that, like all regression models, this analysis helped to uncover significant associations between livelihood strategies and capital assets, but was not a direct test of causal links.

Third, to determine whether food security measured through HFIAS scores responded significantly to the types of livelihood strategies and socio-demographic variables such as the gender of household head, age, household size, number of ill household members, and educational attainment of the household head (objective 3), we ran a generalized linear model using a quasi-Poisson error distribution to account for overdispersion. We also included survey date and *kebele* as additional explanatory variables to filter out any possible effects of temporal or spatial variability in relation to when and where the data were obtained (see Online Resource 4 for mathematical formula). Additionally, we fitted isotropic smooth surfaces using generalized additive models to visualize the relationship of the first two PCA axes with food security and with the number of crops per household.

## Results

### Description of local context

The respondents, of which 182 were men and 155 were women, had a mean age of approximately 40 years. On average, they attended school for between 1 and 6 years. Households had an average of six members (see Table 2 for household characteristics by livelihood strategy). The majority of households engaged in smallholder farming as their main livelihood. The most common livelihood activities involved production of food crops namely maize, sorghum and teff. Barley and wheat were also produced but in lower quantities (Table 3). These food crops were produced mainly for subsistence, with a range of 93–100% of harvest reported as used for consumption. The crops coffee and khat were the main sources of cash. Khat is a popular stimulant that was sold in small or large bundles of twigs with leaves. There were other livelihood activities in the area including the cultivation of home gardens, production of legumes, production of milk, cheese, butter and honey for household consumption and the local market, selling firewood, selling eucalyptus trees, and engagement in farm labor and non-farm labor for wages.

**Table 2:** Household characteristics and capital assets summarized by livelihood strategy

Variables (mean ± standard deviation where applicable)	Three food crops, coffee and khat	Three food crops and khat	Two food crops, coffee and khat	Two food crops and khat	One food crop, coffee and khat
Household characteristics					
Household type (proportion of FHH – female-headed households, MHH – male-headed households)	FHH – 9 MHH – 91	FHH – 8 MHH – 92	FHH – 6 MHH – 94	FHH – 8 MHH – 92	FHH – 7 MHH – 93
Age of household head (yrs)	41 ± 16	40 ± 15	44 ± 16	39 ± 15	41 ± 16
Education of household head (ordinal categories)	1 ± 1	1 ± 1	0.6 ± 0.9	1 ± 1	1 ± 1
Household size (nr)	6.2 ± 2.9	6.5 ± 2.8	6.1 ± 2.5	5.9 ± 2.4	5.8 ± 2.3
Ill health members (nr)	0.3 ± 0.6	0.3 ± 0.5	0.4 ± 0.6	0.4 ± 0.7	0.3 ± 0.5
Capital assets					
Ownership of coffee plot (proportion of yes/no)	Yes – 99 No – 1	Yes – 22 No – 78	Yes – 100 No – 0	Yes – 20 No – 80	Yes – 91 No – 9
Total size of farm fields (ha)	0.9 ± 0.5	1.1 ± 0.7	0.8 ± 0.4	0.7 ± 0.3	0.3 ± 0.3
Sharecropped fields (nr)	1.5 ± 1.3	2.2 ± 1.4	1.6 ± 1.1	1.7 ± 1.3	0.5 ± 0.7
Livestock owned (nr)	3.2 ± 2.6	5.1 ± 4.5	3.6 ± 2.6	4.0 ± 3.1	2.0 ± 1.2
Learn from other farmers (proportion according to frequency)	Never – 35 Rarely – 22 Seldom – 22 Often – 21	Never – 42 Rarely – 17 Seldom – 25 Often – 15	Never – 53 Rarely – 14 Seldom – 20 Often – 13	Never – 60 Rarely – 8 Seldom – 26 Often – 6	Never – 64 Rarely – 11 Seldom – 16 Often – 9
Learn from development agents (proportion according to frequency)	Never – 26 Rarely – 25 Seldom – 37 Often - 12	Never – 46 Rarely – 22 Seldom – 22 Often – 10	Never – 37 Rarely – 21 Seldom – 22 Often – 20	Never – 52 Rarely – 19 Seldom – 21 Often - 8	Never – 23 Rarely – 20 Seldom – 41 Often – 16
Perception of the quality of change in environment (proportion of positive/negative)	Positive – 63 Negative – 37	Positive – 37 Negative – 63	Positive – 54 Negative – 46	Positive – 48 Negative – 52	Positive – 80 Negative – 20
Farm tools owned (nr)	2.1 ± 2.4	1.6 ± 2.0	2.2 ± 2.1	1.2 ± 1.7	1.2 ± 1.6
Access to honey in the forest (proportion of yes/no)	Yes – 31 No – 69	Yes – 27 No – 73	Yes – 26 No – 74	Yes – 16 No – 84	Yes – 23 No – 77
Mobile phone (proportion of yes/no)	Yes – 41 No - 59	Yes – 34 No – 66	Yes – 33 No – 67	Yes – 25 No – 75	Yes – 39 No – 61

For some variables, “nr” means number, for example number of sharecropped fields, or number of livestock owned. For education of household head, “ordinal categories” refer to ordinal categories of educational attainment in which No education = 0, Adult education or special education = 1, Grades 1–6 = 2, Grades 7–12 = 3, and Grades 13 and above = 4

**Table 3:** Main crops, mean harvest (kg) per household, percentage of harvest used for subsistence and percentage of harvest sold. Khat is an important livelihood variable. However, because respondents were unable to give reliable data on quantity of harvest or income due to mechanism of harvest and selling, we used presence-absence data for this variable

Main crops	Mean harvest (kg) per household ± standard deviation	Percentage of harvest used for subsistence	Percentage of harvest sold
Maize	285 ± 459	93	7
Teff	100 ± 153	98	2
Sorghum	84 ± 157	95	5
Barley	11 ± 37	99	1
Wheat	10 ± 39	100	0
Coffee	170 ± 320	23	77
Khat	*131 households had khat*	*Some khat was used by the households*	*Most khat was produced for the local market*

Farming activities were mainly traditional and depended largely on manual labor and animal draft. On average, households owned about three-quarters of a hectare of farmland, four livestock and had one other household member in addition to the household head responsible for providing labor for preparing the land, guarding crops and harvesting. Common livelihood problems such as lack of farmland, livestock and labor were typically addressed through sharecropping arrangements. An average of two fields for each household were sharecropped fields. Most households had limited connection to markets either for selling their produce or purchasing goods. At the *kebele* level, there were two types of markets. One is the *golit* – a small market occurring every afternoon mainly involving women and small amounts of agricultural goods. The *gaba* is a larger market occurring once a week, involving both men and women. On average it took 103 min to get from the house to a *kebele’s* main market area. Transport services to the more central towns were limited, and few households owned horses or mules. Access to credit was also limited. Some households used informal credit channels such as borrowing coffee or cash from neighbors, friends or kin to address shortfalls.

### Typologies of livelihood strategies

**Fig. 1:**
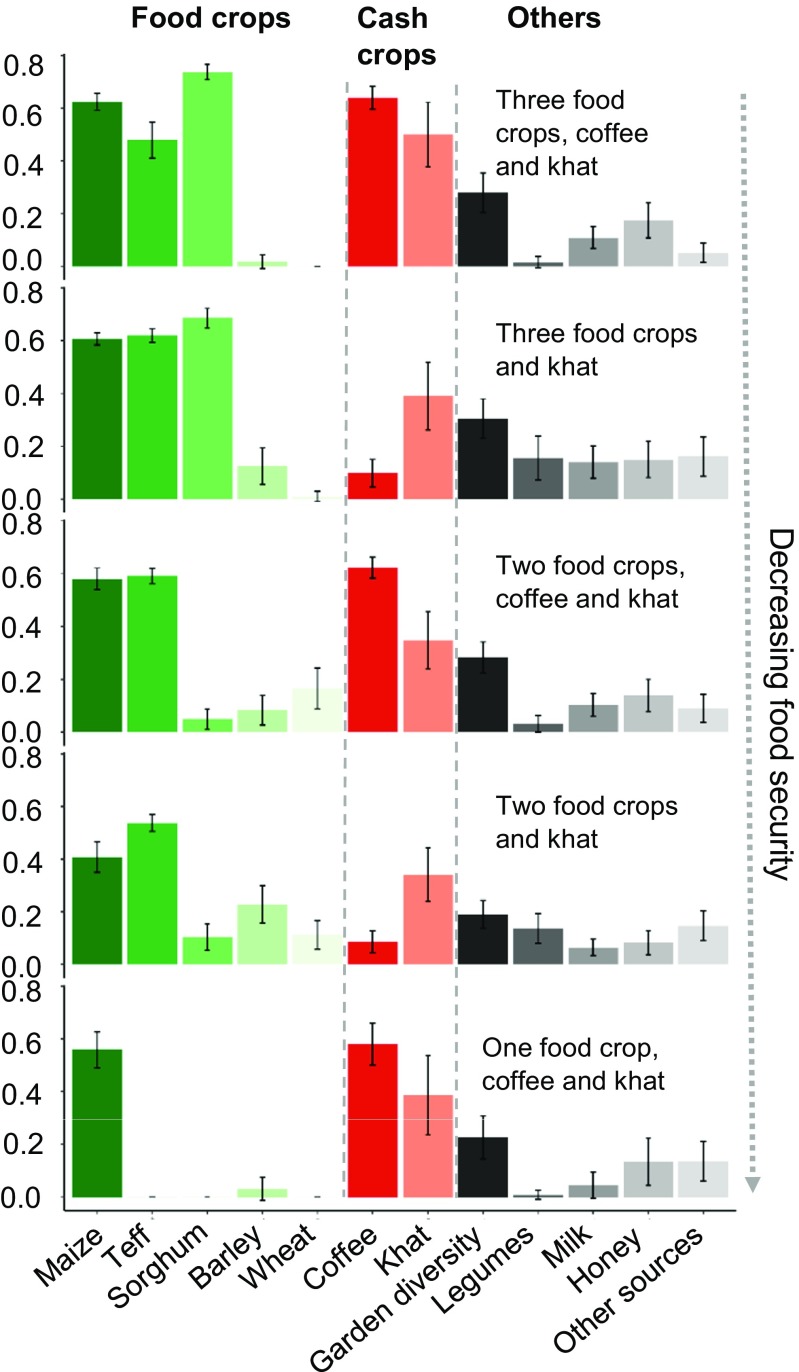
Livelihood profiles. The x-axis shows livelihood activities in the study area. The y-axis indicates livelihood components. Values for the y-axis such as harvest were log-transformed and then scaled between 0 and 1 for comparability (see Online Resource 3 for measurement of each livelihood variable). Error bars indicate 95% confidence intervals

Different combinations of cash crops and food crops distinctively defined the livelihood strategies of households. Households typically produced multiple crops, three on average. Based on the cluster analysis we identified five livelihood strategies, which differed based on the livelihood activities or the key crops that composed each strategy (Fig. 1; also see Online Resource 5 for dendrogram). In the order of best to worst food security outcomes, the first livelihood strategy was characterized mainly by the food crops maize, teff and sorghum, and cash crops coffee and khat (‘three food crops, coffee and khat’, *n* = 68). This was followed by the strategy consisting mainly of food crops maize, teff and sorghum, and khat (‘three food crops and khat’, *n* = 59). These two strategies with the best food security outcomes notably included three food crops, with the difference of the first strategy having two cash crops and the second having only one cash crop.

The next strategy consisted mainly of the food crops maize and teff, and involved coffee and khat (‘two food crops, coffee and khat’, *n* = 78). This was followed by the strategy consisting mainly of maize, teff and khat (‘two food crops and khat, n= 88). The final livelihood strategy with the lowest food security had only maize as food crop, and coffee and khat (‘one food crop, coffee and khat’, *n* = 44). Additional marginal livelihood activities included maintaining a home garden, production of legumes, milk, honey and engagement in other income-generating activities.

**Fig. 2:**
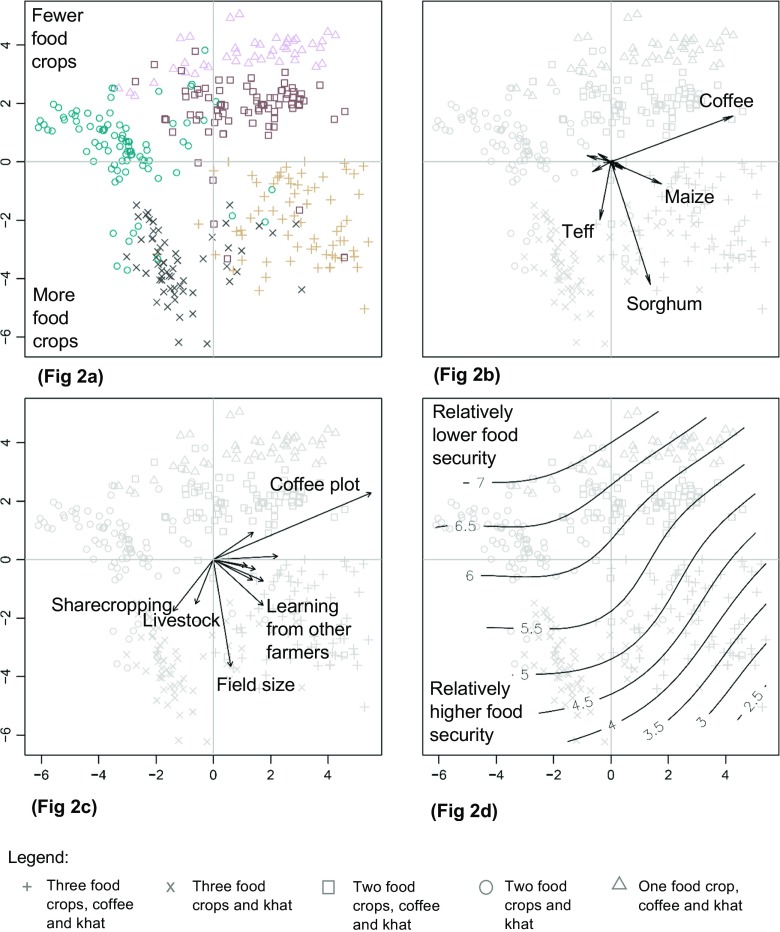
Ordination plots of livelihood strategies with associated capital assets and food security outcomes. Underlying all four panels are the combined principal component analysis (PCA) and the cluster analysis of livelihood variables with each data point representing a household and a corresponding livelihood strategy indicated by a symbol. The x-axis always depicts the first principal component (26% explained variation) and the y-axis the second principal component (23% explained variation). **a** Distribution of households by livelihood strategies in the ordination space of the PCA. **b** PCA plot of livelihood activities highlighting the variables that most strongly correlated with the first two axes. Longer arrows suggest stronger correlations with PCA axes. **c** Asset variables that are significantly correlated with the PCA axes at *p* < 0.01 (permutation test). Longer arrows also suggest stronger correlations with PCA axes. **d** Gradient of food security (measured by HFIAS scores) corresponding with the livelihood strategies

Clustering of households according to livelihood strategies corresponded well with the PCA ordination plot suggesting robustness of groupings (Fig. 2a). Each point in Fig. 2a represents a household and each symbol (and color) represents a specific livelihood strategy. The nearness of households with the same livelihood strategy in the PCA plot indicates consistency of groupings between cluster analysis and PCA. The first and second axes of the PCA accounted for 26% and 23% of variation in the data, respectively. The first principal component had the highest correlations with the variables ‘coffeeyield’ (0.85), ‘maizeyield’ (0.35), and ‘sorghumyield’ (0.27). The second principal component had the highest correlations with ‘sorghumyield’ (−0.84), ‘teffyield’ (−0.40) and ‘coffeeyield’ (0.31) (Table 4). These correlations in the PCA indicated by the longer arrows (Fig. 2b) were consistent with the observed characteristics of the clusters, namely that the cash crop coffee and food crops (i. e. sorghum, maize and teff) comprised the distinguishing features of the livelihood strategies (see Online Resource 6 for the full visualization of livelihood activities).

**Table 4:** Livelihood activities and PCA loadings

Livelihood variables	Principal component 1	Principal component 2
maizeyield	0.35	−0.15
teffyield	−0.077	−0.40
sorghumyield	0.27	−0.84
barleyyield	−0.17	0.042
wheatyield	−0.089	0.056
coffeeyield	0.85	0.31
khat	0.020	−0.0028
gardendiversity	0.079	−0.051
legumes	−0.13	−0.068
milk_liter	0.028	−0.054
honey_kg	0.10	−0.045
oth.income	−0.022	0.0022

### Associations between capital assets and livelihood strategies

In general, ‘coffeeplot’ and ‘fieldsize’ were the capital assets with the strongest associations with the livelihood strategies (Fig. 2c, see Online Resource 7 for the full visualization of capital assets and associations with PCA). This suggests that the ability of households to undertake the production of food crops and cash crops was strongly associated with their access to coffee plot and the size of their farmland. This was consistent with the multinomial logistic regression, which tested for relationships between livelihood strategies and capital assets and identified significant relationships with ‘fieldsize’ (*p* < 0.001), ‘coffeeplot’ (p < 0.001), ‘livestock’ (*p* = 0.005), and ‘farmtools’ (*p* = 0.03) (Table 5).

**Table 5:** ANOVA table of multinomial logistic regression applied to capital asset variables against livelihood strategies

Capital assets	LR Chisq	Degrees of freedom	*P* value
livestock	14.72	4	0.0053**
mobilephone	1.87	4	0.76
farmtools	11.07	4	0.025*
learn_DAs	5.18	4	0.27
learn_farmers	5.94	4	0.20
sharecrop	7.58	4	0.11
coffeeplot	227.10	4	<0.001***
envichange	6.26	4	0.18
accesshoney	5.13	4	0.27
landrights	1.37	4	0.85
fieldsize	77.49	4	<0.001***

Significant codes: 0 ‘***’ 0.001 ‘**’ 0.01 ‘*’ 0.05 ‘’ 1

In Fig. 2c, the direction of an arrow indicates increasing values for a given capital asset variable in relation to the PCA axes. The length of an arrow indicates the strength of correlation. The plot indicates that capital assets differed in their association with the livelihood strategies (*p* < 0.01). The strategies involving three food crops were associated with having larger fields. The strategy ‘three food crops, coffee and khat’ had higher access to a range of capital assets. For example, they were more involved in learning with other farmers through informal exchange of information and knowledge. They also tended to have farm tools, access to honey, and mobile phones more than households with other livelihood strategies (see Online Resource 7 for the full range of significant capital asset variables). The livelihood strategy ‘three food crops and khat’ (lower left hand corner) had higher engagement in sharecropping and had more livestock. The strategies ‘two food crops, coffee and khat’ and ‘one food crop, coffee and khat’ were strongly characterized by ownership of coffee plots (upper right hand corner).^2^ Households undertaking these strategies also learned farming techniques through the government’s development agents and had the perception that the condition of the environment had been improving. The strategy ‘two food crops and khat’ (upper left hand corner) did not show strong positive association with any particular capital asset.

In summary, livelihood strategies with coffee were associated with having access to coffee plots. Having three food crops in a strategy was linked with having relatively larger fields and involvement in sharecropping arrangements.

### Food security and explanatory variables

Food security, as measured by HFIAS scores, was significantly associated with the types of livelihood strategies at *p* = 0.03 (Tables 6 and 7). Moreover, Fig. 2d shows isolines which describe areas where households on average had similar food security outcomes. This visualization shows that households undertaking livelihood strategies with a higher number of food crops (lower right hand corner) were more food secure than those with a lower number of food crops (upper left hand corner).

**Table 6:** Independent variables tested against household food insecurity access scale (HFIAS) score, a measure of household food security, and their expected relationships with food security. Low HFIAS scores mean households are more food secure, while high scores mean households are less food secure

Independent variables	Type of variable	Expected relationships	References
Livelihood strategy	Categorical	Households with more diverse livelihood strategies will tend to be more food secure.	Pellegrini and Tasciotti 2014
Gender of household head	Categorical	Male headed-households will tend to be more food secure due to systematic gendered privilege.	Quisumbing et al. 2015
Age of household head	Discrete	Households with older household head will tend to be less food secure due to reduction in available labor.	Zakari et al. 2014
Education of household head	Ordinal	Households with more educated household head will tend to be more food secure due to better knowledge, connections, and opportunities.	Ogundari 2014
Number of ill household members	Discrete	Households with more ill household members will tend to be less food secure because of reduction in available farm labor and/or medical expenses.	Espitia et al. 2018
*Kebele*	Confounding/categorical	*Kebele* will have no significant effect	–
Survey date	Discrete	Survey date will have no significant effect	–

**Table 7:** ANOVA table of generalized linear model. The response variable is household food security measured through household food insecurity access scale (HFIAS) scores. The independent variable livelihood strategy is a categorical variable that represents the five livelihood strategies identified (i. e. ‘three food crops, coffee and khat’, ‘three food crops and khat’, ‘two food crops, coffee and khat’, ‘two food crops and khat’, and ‘one food crop, coffee and khat’)

Independent variables	Sum of squares	Degrees of freedom	F value	P value
Livelihood strategy	25.82	4	2.66	0.032*
Gender of household head	11.68	1	4.81	0.029*
Survey date	1.76	1	0.73	0.39
Age of household head	1.52	1	0.62	0.43
Educational attainment of household head	24.67	1	3.39	0.018*
Household size	0.41	1	0.17	0.68
Number of ill household members	0.58	1	0.24	0.63
*Kebele*	22.70	5	1.87	0.099
Residuals	750.21	309		

Significant codes: 0 ‘***’ 0.001 ‘**’ 0.01 ‘*’ 0.05 ‘’ 1

**Fig. 3:**
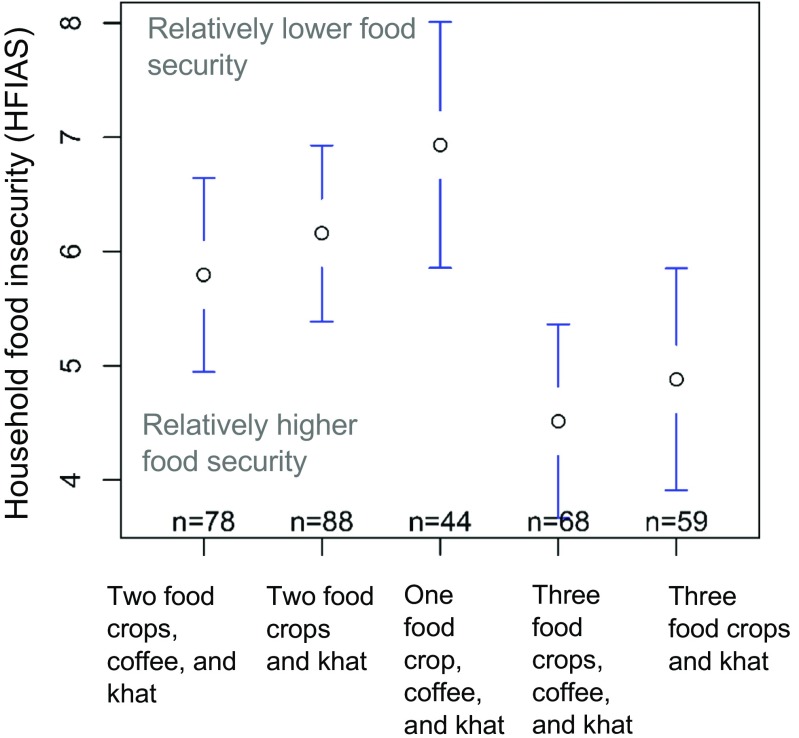
Plot of means of HFIAS scores by livelihood strategy. Error bars indicate standard error

Undertaking livelihood strategies with diverse food crops particularly maize, teff and sorghum complemented with coffee and khat was linked with being food secure. Having only maize, or maize and teff, even in combination with coffee and khat, was associated with lower food security. Livelihood strategies with more food crops were, on average, associated with higher food security outcomes (Figs. 2d, 3 and Online Resource 8). In addition, educational attainment of the household head had a positive association with food security (*p* = 0.02). Gender of household head was also significantly associated (p = 0.03). Male headed- households tended to have better food security than female-headed households. Other explanatory variables tested in the model, including survey date, age of household head, household size, number of ill household members, and *kebele* did not show any significant association.

## Discussion

Our study identified five types of livelihood strategies following a gradient in composition of food and cash crops. Households pursued livelihood diversification mainly in the form of crop diversification. This is somewhat at odds with the trajectories envisaged in agricultural policies in Ethiopia and other developing countries, which prioritize production of cash crops (and food crops for commercial purposes) as a pathway for development and food security. The dissimilarity between these identified local livelihood strategies and the strategies endorsed and supported by policies is notable (Arce 2003) because evidence on the food security benefits of livelihood shifts to cash crop production has been varied and conflicting. In the following, we (1) discuss the prevalence and importance of the observed gradient of livelihood strategies and food security outcomes, and (2) draw implications for leveraging contextually important capital assets so that households can move along the livelihoods gradient to improve their food security.

### Gradient of livelihood strategies and food security

Ellis (2000) discussed the importance of livelihoods diversification in a context characterized by precarious conditions and a need for survival. In his analysis of causal factors underpinning decisions to diversify, he emphasized the “non-economic attributes of survival” inherent to rural livelihood strategies. We conjecture that for households in southwest Ethiopia, the feature of diverse crops in the livelihood strategies may be motivated not so much by economic profitability and capital asset accumulation but by the basic need to ensure households’ direct access to food.

The observed importance of diverse food crops in local livelihood strategies is consistent with the findings of Fafchamps (1992), who observed the critical importance of staple consumption for survival. Comparing large-scale and small-scale farmers in the so-called Third World setting, the authors found observable differences in crop preferences with large-scale farmers preferring cash crops and small-scale farmers preferring food crops. For small-scale farmers, food self-sufficiency through food crop production was found to be the best approach for assuring food security, even when food markets were present. A recent study in the Eastern Cape, South Africa, also found that household food production for the purpose of household consumption resulted in lower levels of hunger. Although wage income was considered important, household food production was critical for addressing the immediacy of food security concerns (Rogan 2018). Similarly, in our study, cash crops played an important role in income generation. Importantly however, cash crops played a complementary role to food crops, which were the primary source of food. With combinations of diverse food and cash crops, households in southwest Ethiopia were able to take advantage of what Ellis (2000) termed “complementarities between crops”. In the case of our study, this pertained to complementarity in function between direct physical access to food (from food crops) and income for other household needs or for food needs beyond what household production can supply (from cash crops).

Our study showed that combinations of food crops and cash crops, particularly diverse food crops, were important for the food security of households. Comparing the two livelihood strategies with the strongest contrast in food security status (i.e. ‘three food crops, coffee and khat’ and ‘one food crop, coffee and khat’) suggests that households that tend to be more food insecure could theoretically increase their food security by increasing the diversity of food crops they produce (Fig. 2d). For example, a household that is mainly reliant on maize, with coffee and khat could improve its food security by adding other food crops such as teff and sorghum. This underscores a pathway to food security that is distinct from the market-oriented pathway of the Ethiopian agricultural policy. It is a pathway that emerges from the semi-subsistence production and consumption practices of the households in the area. In a study in Malawi, Radchenko and Corral (2018) found varied effects of agricultural commercialization on nutritional outcomes for households in different tiers of the population – benefitting some and harming others. Malawian households were likely to focus on food crops when they expected food insecurity and malnutrition. However, under conditions of weaker market barriers, households were likely to choose cash crops. These findings may also explain the preponderance of diverse food crops in southwest Ethiopia, which has been similarly characterized by seasonal food insecurity (Ethiopia CSA and WFP 2014) and limited market access. Findings by other researchers have also identified market access and infrastructure (e.g. transportation) as important contextual factors that influence the choice and outcomes of crop production (Fafchamps 1992; Radchenko and Corral 2018). A limitation of our household level investigation was that we did not include a systematic analysis of these contextual factors and the logic underpinning households’ strategies in view of these factors. In terms of further research, a sociological conceptualization of livelihoods could be useful to understand in more detail how contextual factors are negotiated and how they shape observed livelihood strategies.

### Supporting local livelihoods: leveraging contextually important capital assets

Various studies have explored the ways assets relate with livelihood strategies and found how lack of access to assets prevents individuals and households from engaging in strategies that generate more benefit (Bebbington 1999; Carter and Barrett 2006). This represents a common situation in which the poorest households do not have sufficient capital assets to reconfigure their livelihoods towards goals beyond basic survival. In our study area, households that had larger areas of farmland were able to engage in the strategy that had high diversity in food and cash crops, which subsequently generated better food security outcomes. They also had access to a wider range of capital assets. Supporting households to pursue livelihood strategies with diverse food and cash crops thus should be cognizant of the need to address shortages in capital assets.

Most notably, the field size that households were entitled to, turned out to be strongly correlated with livelihood strategies. Presently, land ownership in Ethiopia rests with the government and individuals hold usufruct rights to land. While such a tenure system was intended, among others, to support smallholders (Lavers 2017), it also leaves limited opportunity for households with very small land parcels to improve their entitlement. Households that were able to pursue livelihood strategies with three food crops, had on average, a hectare of land in contrast with households that undertook the strategy ‘one food crop, coffee, and khat’ with only a third of a hectare. The challenge of small land holdings is likely to further increase due to rapid population growth, with smaller parcels of land being inherited by each subsequent generation (Gebrehiwot et al. 2016). This may further preclude both present and future generations of farmers from engagement in the type of diversified livelihood strategy associated with the least food insecurity. Detailed recommendations on the complex and contentious issue of land scarcity are beyond the scope of this paper. At a basic level, however, and in view of land-grabbing in various parts of Ethiopia (Ango 2018), opening space for debate at the policy level, and exploring options for land sufficiency at the household level should at least be taken up; possibly alongside culturally appropriate efforts to address population growth. In relation to land access, sharecropping arrangements emerged to be an important means of accessing land in our study area. Households that were engaged in livelihood strategies involving one to two food crops and had lower food security, were not as much engaged in sharecropping as those producing three food crops. Investigating the factors that underlie Ethiopian sharecropping arrangements including input contribution, risk distribution, and benefit distribution may be an important step for understanding and exploring contextually suitable options for strengthening and embedding equity in these arrangements.

Furthermore, food security was not only influenced by livelihood strategies, but also by other household characteristics such as gender and educational attainment of the household head. Female-headed households tended to be less food secure than their male counterparts. This is in line with findings from gender and development research that examined systematic inequality around access and control of capital assets (Quisumbing et al. 2015) and decision-making processes (e. g. Sumner et al. 2017) causing serious disadvantage among female heads of households. In other parts of Ethiopia, women’s social ties have been found to be less linked to the formal economy (Torkelsson 2007); and they have less control and access to important assets such as land and labor (Quisumbing et al. 2015). Improvements to gender equality thus emerge as an important precondition for achieving food security (Njuki et al. 2016).

Unlike other studies, we found no significant relationship between household size and food security. This could be because, in this context, household size is important for labor, but may also be negatively related to food availability because of more household members to feed (e. g. Feleke et al. 2005; Akinboade and Adeyefa 2018). Age of household head was similarly not significantly related to food security. Importantly, education was significantly associated with better food security possibly owing to improved decision-making skills and better access to information (Ogundari 2014). In summary, our findings thus suggest that access to land, fair sharecropping arrangements, gender equality, and education are foundational requirements for food security in southwest Ethiopia.

## Conclusion

Based on the observed farming practices in the study area, diversified production of both food and cash crops should be encouraged to improve food security. Policies that seek to promote food security of smallholder farming households would do well to recognize and support the complementarities between food crops and cash crops rather than impose a narrowly framed economic growth narrative that can potentially erode these complementarities. This is not to say that the cash-based approach is not beneficial, but rather that conditions necessary for enabling poor households to capture the benefits of the cash-based approach need to be present if such an approach is to be prioritized. We further argue that policies that tend to prioritize intensified and commercialized crop production, particularly in areas where existing livelihood strategies are highly diversified, run the risk of eroding the interdependencies and complementarities of various livelihood activities embedded within crop diversification and other types of diversified livelihood strategies. Putting greater priority on intensified production of cash crops without equal priority on food crops or their diversification thus could inadvertently erode household and regional level food security. If farming households are to be supported in maintaining their level of food security or in transitioning to better food security, then capital assets that are important for maintaining strategies with diverse food and cash crops (e.g. three food crops, coffee and khat) should be given priority attention. Supporting farming households to shift towards livelihood strategies associated with better food security outcomes should consider the elements embedded in households’ current strategies and support them in accessing those capital assets they need to expand the sphere of their means and goals (Rakodi 1999).

## Electronic supplementary material

ESM 1 (PDF 154 kb)

ESM 2 (PDF 363 kb)

ESM 3 (PDF 85 kb)

ESM 4 (PDF 156 kb)

ESM 5 (PDF 155 kb)

ESM 6 (PDF 258 kb)

ESM 7 (PDF 166 kb)

ESM 8 (PDF 261 kb)

## Footnotes

1 Analysis involved continuous harvest data for all main crops except khat for which we were limited to using presence-absence data due to a lack of reliable data on both harvest and income. We ran PCA analysis without the variable khat to check robustness of results. We found that results with and without khat were very similar (correlation in a symmetric Procrustes rotation of 0.9962). This suggests that including khat as a binomial variable did not unduly influence the results.

2 The widespread practice of sharecropping, including in coffee production, meant that there were households that harvested coffee but did not own coffee plots. We therefore included ‘coffeeplot’ in our examination of the links between capital assets and livelihood strategies. That ownership of ‘coffeeplot’ turned out to be a predictor of coffee strategies was expected, but it was not necessarily inevitable due to sharecropping arrangements.
