# Supplementary material for: Livelihood strategies, capital assets, and food security in rural Southwest Ethiopia
Source: Food Secur. 2019 Jan 24;11(1):167–81. doi: 10.1007/s12571-018-00883-x (PMC6411135; doi:10.1007/s12571-018-00883-x)
Supplement: Supplementary file 1 — (PDF 154 kb) [file 12571_2018_883_MOESM1_ESM.pdf]

Online Resource 1 *Kebeles* comprising the study area, corresponding altitude, and forest cover. The primary altitude range within which coffee is grown in this region is below 2000 m above sea level (asl) (Lemessa, Hambäck, and Hylander 2015).

Kebele	***Woreda*/ district**	**Altitude range (m asl)**	**Median altitude (m asl)**	**Forest cover (%)**	**Total number of households**	**Number of households included in analysis**
Borco Deeqaa	*Geeraa*	1500 - 2180	1768	72	802	68
Kellaa Hareerii	*Geeraa*	1600 – 2900	2274	79	322	28
Qudaa Qufii	*Gumaay*	1600 – 2210	1839	39	512	41
Barahaa Waraango	*Gumaay*	2120-2600	2248	0	1222	98
Gidoo Barii	*Saxammaa*	1780 - 2230	2045	32	691	59
Difoo Maanii	*Saxammaa*	1520 - 1980	1745	33	532	43

Aisa O. Manlosa, Jan Hanspach, Jannik Schultner, Ine Dorresteijn, and Joern Fischer
Corresponding author: Aisa O. Manlosa, Faculty of Sustainability, Leuphana University Lüneburg, aisamanlosa@gmail.com
