# Supplementary material for: Livelihood strategies, capital assets, and food security in rural Southwest Ethiopia
Source: Food Secur. 2019 Jan 24;11(1):167–81. doi: 10.1007/s12571-018-00883-x (PMC6411135; doi:10.1007/s12571-018-00883-x)
Supplement: Supplementary file 2 — (PDF 363 kb) [file 12571_2018_883_MOESM2_ESM.pdf]

Online Resource 2 Survey questionnaire

Interviewer ID: ______________________ *Date survey was conducted:*______________________

Surveyed during Pilot Study? O Yes O No

Note to interviewers: The contents that are italicized are for you, they are not to be asked or read aloud to the respondents.

(If surveyed in pilot, follow this spiel.) I am/We are part of the team from Addis Ababa University and Leuphana University in Germany studying food security and biodiversity. Some of our colleagues may have visited you some weeks or months ago and explained what the project is about. You may remember that they asked you if you were willing to be part of this study and you agreed. I would like to thank you for agreeing to be part of this. I am here because I wanted to talk with you about your livelihood, the diversity of crops you use, and the food security condition of your household. I would like to have a better understanding of the relationships between these. If it is alright with you, I would like to ask you to be part of this survey. It will include some general questions about your household, about your livelihoods, crops you grow, and the food items that your household uses. The whole survey will take about an hour or a little bit more. If there are questions that you don't want to answer, you are free not to answer them. Also, you are free to end this interview anytime you wish to, although I would really appreciate hearing your thoughts about all the questions I have. I would like to assure you that we will not use your name or the name of your community in any future publication coming out of this study. We cannot say that you or your community will directly benefit from the results of this study, but the study can generate information that may help us understand issues of food security and livelihoods better. Do you have any questions before we start? If not, we can start.

(If not surveyed in pilot, use this spiel instead.) I am/We are part of the team from Addis Ababa University and Leuphana University in Germany studying food security and biodiversity. The student doing this research seeks to understand how livelihoods of people affect the food security of their households, and how biodiversity also affects food security. I would like to gather information from six kebeles in this region, and your household is one of those that were randomly chosen *(explain a bit more about why selection was random and how they were randomly selected)*. If it is alright with you, I would like to ask you to be part of this survey. It will include some general questions about your household, about your livelihoods, crops you grow, and the food items that your household uses. The whole survey will take about an hour or a bit more. If there are questions that you don't want to answer, you are free not to answer them. Also, you are free to end this interview anytime you wish to, although I would really appreciate hearing your thoughts about all the questions I have. I would like to assure you that we will not use your name or the name of your community in any future publication coming out of this study. We cannot say that you or your community will directly benefit from the results of this study, but the study can generate information that may help us understand issues of food security and livelihoods better. Do you have any questions before we start? If not, we can start.

Witness (*write who else is present as witness that the respondent gave his/her consent to do the survey*):
____

Aisa O. Manlosa, Jan Hanspach, Jannik Schultner, Ine Dorresteijn, and Joern Fischer
Corresponding author: Aisa O. Manlosa, Faculty of Sustainability, Leuphana University Lüneburg, aisamanlosa@gmail.com

1. **Household Profile** (*For households that were surveyed during the pilot study, some of the information in this section have already been collected. They are marked with asterisks. Do not repeat the questions, but try to confirm their previous answers by saying "You had been asked some questions in a survey by other team members of this research a few months ago… and just mention some of the answers they already gave and ask if these are right.)*

1.1 Name (***Do not ask***, *only write if the person introduces himself or herself)*:	1.2 Sex: O Female O Male	*1.3 Age:
*1.4 Religion:<br>O Muslim O Orthodox<br>O Protestant O Catholic<br>O Others, _____________	*1.5 Marital Status:<br>O Single O Married<br>O Divorced O Widowed	1.5.1 If married, type of household:<br>O Monogamous<br>O Polygamous
1.5.2 If marriage is polygamous, how many wives does the household head have:<br>O 1 O 2 O ________	*1.6 Household Size: ________<br>*Note: A household is made of people that live together in one house and share a meal.*	*1.7 Number of dependent children: ______________<br>*(Children that don't have spouses and kids of their own and no earning yet.)*
*1.8 What grade of education did you complete?<br>________________________<br>And how about your spouse?<br>________________________	*1.9 When did you settle in this particular spot?<br>O _________ (Ethiopian year)<br>O A long time ago but I cannot recall<br>O Always been here<br>O Born here, left, and came back on ________________	*1.10 If respondent was not born in the kebele, ask from where he or she was:<br>O Within Oromia<br>O Other: _____________
*1.11 Did your parents live in this region?<br>O Yes<br>O No<br>If no, from where?<br>_______________________	1.12 Do you have children who dropped out of school within the last three years?<br>O Yes<br>If yes, how many: ______<br>O No	1.13 Is there any member of this household who has been repeatedly or continuously ill within the last three years? *(ill for a long time, for example continuously sick for nearly one month)*<br>O Yes<br>If yes, how many: _____<br>O No
*1.14 How many farm fields do you farm in? *(including home garden, crop fields owned, and fields where person is sharecropping)*<br>_______________________	1.15 What is the area of land your household farms?<br>Home garden: ___________<br>Sharecropping: __________<br>*Field 1: _________________<br>*Field 2: _________________<br>*Field 3: _________________<br>*(in hectare or oxen days but be sure to ask how many oxen days is equivalent to 1 ha; for fields 1-3 or more, specify crops planted. For households included in pilot study, skip the fields with asterisk)*	1.16 How far is the nearest source of drinking water? *(Use time required to get there.)*<br>_______________________
1.17 What type of toilet does your household have?	1.18 (Do not ask, infer if possible.)	

Aisa O. Manlosa, Jan Hanspach, Jannik Schultner, Ine Dorresteijn, and Joern Fischer
Corresponding author: Aisa O. Manlosa, Faculty of Sustainability, Leuphana University Lüneburg, aisamanlosa@gmail.com

O Pit latrine<br>O Hole<br>O We go outside<br>O Others _________________	Ethnicity:<br>O Oromo<br>O Amharic<br>Others:__________________	

Part 2. Livelihood Strategies and Crop Diversity

(Recall time for questions in this part: present year)

2.1 What are the crops that you produce? *(Check those that apply and ask the next questions. Write down if there are others.)*

***Crops produced**	***Improved or not?<br>0 – No<br>1 – Yes**	***Quantity of average harvest before consumption and selling *(either in kilograms or quintal)***	**Indicate if from sharecropping farm (SF), own farm (OF), homegarden (H), and forest (F)**	**How much is consumed?**	**How much is sold?**	**Where sold?<br>trader (T)<br>market (M)<br>community (C)**	**What is the cash income estimate from selling?**
O Coffee							
O Maize							
O Teff							
O Sorghum							
O Wheat							
O Barley							
O Khat							

2.2 Are there other plants that women specifically produce and earn from?

**Plants produced**	**Quantity of average harvest**	**How much is sold?**	**What is the cash income estimate from selling?**
O Cabbage			
O Onion			
O Pepper			
Others:			

2.3 Are there other farm, off-farm, or non-farm activities that members of the household engage in which generate cash or other forms of income (e. g. free use of oxen, free use of land, free use of farming equipment, free food)? *(Check those that apply and ask follow up questions.)*

Aisa O. Manlosa, Jan Hanspach, Jannik Schultner, Ine Dorresteijn, and Joern Fischer
Corresponding author: Aisa O. Manlosa, Faculty of Sustainability, Leuphana University Lüneburg, aisamanlosa@gmail.com

*Other income-generating activities	Who are involved? (husband (h), wife (w), children (c))	What is the proportion sold? / How much is the income?
O Livestock: selling milk		/
O Livestock: selling meat		/
O Livestock: selling live animal		/
O Beekeeping/selling honey		/
O Making and selling handicraft		/
O Operating a store/small business/petty trade		/
O Wage labor in other farms		/
O Non-farm wage labor (e. g. construction work)		/
O Sell of firewood/charcoal		/
O Remittance		/
O Others, write below		/
		/
		/
		/
**2.3a Are you collecting these materials? Check if yes.[1]**		
	**How much?**	**Sources (OF – own farm or F - forest)**
O Honey	Last harvest (kg):	
O Eucalyptus	Nr. of trees standing:	
O Wood for plow, hoe, axe, spade	Nr. Of plows __, hoes __, axes __, spades__ last year	
O Fuel wool	Nr of loads per week:	

2.4 In some households, some crops are set aside for consumption. *(*You mentioned some crops which you consume..*) Are there other plants in your home garden or farm fields which you also collect and consume as food?*

Other plants eaten	Frequency of eating (S – when it is the season; AY – it is available all year round and we eat when available; NOF – it is available all year round, but we only eat it when there is no other food to eat)
O Avocado	
O Mango	
O Banana	
O Taro	
O Enset	
O Anchote	
O Beans	
Others:	

2.5 What is the proportion of the total cash income of your household that is spent on food? _________

[1] Ecosystem bundle question.

Aisa O. Manlosa, Jan Hanspach, Jannik Schultner, Ine Dorresteijn, and Joern Fischer
Corresponding author: Aisa O. Manlosa, Faculty of Sustainability, Leuphana University Lüneburg, aisamanlosa@gmail.com

2.6 How does your household get majority of its food during the different seasons in a year? *(Place the letter of choice in the table below. It is possible to indicate two answers for every season.)*

A. We buy most of our food.

B. We produce most of our own food.

C. We borrow from neighbor like when we don't have injera for today, we borrow a little and pay tomorrow.

D. We get most of our food from exchange of products with others.

E. We receive most of our food from others.

**Bira**	**Bone**	**Arfasa**	**Gana**

Part 3. Capital Assets

In this next part, we will talk about the resources that you are able to use for your livelihood.

3.1 Are there other members in your household who are able to help you work in your farm plot?

O Yes   If yes, how many? ________   O No

3.2 Are you able to learn new farming techniques from DAs, extension workers, or NGO programs?

O Yes   If yes, how often?   O Rarely   O Seldom   O Often   O Always

O No

3.3 Are you able to learn new farming techniques from fellow farmers?

O Yes   If yes, how often?   O Rarely   O Seldom   O Often   O Always

O No

3.4 Do you have access to information about new technologies and market prices of agricultural goods?

O Yes   If yes, how often?   O Rarely   O Seldom   O Often   O Always

O No

3.5 Are you an active member of a farming organization or a seller's association?

O Yes   O No

3.6 If you want to invest on your livelihood, are there people (e. g. relatives, neighbors, friends) or organizations that you can borrow money from?

O Yes   If yes, from who? ____

O No

3.7 If you encounter problems in your livelihood such as pest infestation, are there people (e. g. relatives, neighbors, friends) or organizations that you can turn to for help?

Aisa O. Manlosa, Jan Hanspach, Jannik Schultner, Ine Dorresteijn, and Joern Fischer
Corresponding author: Aisa O. Manlosa, Faculty of Sustainability, Leuphana University Lüneburg, aisamanlosa@gmail.com

O Yes If yes, who or what organization? ___________________________________

How do they help?
____

O No

3.8 If there is a shortage of cash earning or of food in your household, are there people (e. g. relatives, neighbors, friends) or organizations that you can turn to for help?

O Yes If yes, who or what organization? ___________________________________

How do they help?
____

O No

3.9 Do you use natural resources such as forests and water?

O Yes

O No

3.10 Are you able to participate in activities related to making decisions for using the forests and water?

O Yes If yes, do you think you are able to say what you want to say? _______________________

O No

3.11 Do you think there have been changes in the quality of the natural resources nearby such as forests, water, and soil?

O Yes If yes, how has it changed? O Become better O Become worse

O No

3.11a How is the fertility of your soil without fertilizers? O good O medium O bad

3.12 Do you think that changes in natural resources nearby affect your livelihood or the availability of food for the household?

O Yes O No

3.13 How far is the nearest market where you can sell your products? __________________________

How about the nearest market where you can buy food and other household needs? __________

3.14 What is the status of your ownership of land?

A. I own a certificate.

B. I received it as inheritance.

C. Others: ____

3.15 Does your household own any of the following items? Can you tell me how many of each your household owns?

Aisa O. Manlosa, Jan Hanspach, Jannik Schultner, Ine Dorresteijn, and Joern Fischer
Corresponding author: Aisa O. Manlosa, Faculty of Sustainability, Leuphana University Lüneburg, aisamanlosa@gmail.com

**Physical resources**	**Quantity/number owned**	**If you don't own these, can you access them through people you know? Write 0 for No, and 1 for Yes.**
*Oxen		
*Cow		
*Cattle (also indicate how many are for beef fattening)	( )	
*Goats		
*Sheep		
*Horse		
*Chicken		
*Mule		
Non-mechanized farm equipment (e. g. machete, hoe, plow made of wood and pulled by oxen)		
Mechanized farm equipment (e. g. tractor)		
Vehicle (e. g. motorbike, bicycle)		
Cellphone		

Part 4. Household Food Security

	**No**	**Yes**	**Rarely**	**Sometimes**	**Often**
4.1 In the period June-August, did you worry that your household would not have enough food?	O		O	O	O
4.2 In the period June-August, have you ever had to eat enset or a food you did not like because there was nothing else to eat?	O		O	O	O
4.3 In the period June-August, did you or any household member have to eat a smaller meal than you felt you needed because there was not enough food?	O		O	O	O
4.4 In the period June-August, did you or any household member have to eat fewer meals in a day because there was not enough food?	O		O	O	O
4.5 In the period June-August, did you or any household member go to sleep at night hungry because there was not enough food?	O		O	O	O

Part 5. Household Dietary Diversity

5.1 What foods have you eaten in the last one week?

	**Food Items**	**Approximately how often did you eat these last week? (Rarely – one time last week, Sometimes – 2-4 times last week, Often – 5-7 times last week)**
Breakfast		

Aisa O. Manlosa, Jan Hanspach, Jannik Schultner, Ine Dorresteijn, and Joern Fischer
Corresponding author: Aisa O. Manlosa, Faculty of Sustainability, Leuphana University Lüneburg, aisamanlosa@gmail.com

Lunch		
Dinner		

5.2 Can you recall what other foods in addition to what you just identified you ate last week?

**Other foods eaten**	**Approximately how often did you eat these last week? (Rarely – one time last week, Sometimes – 2-4 times last week, Often – 5-7 times last week)**
O Eggs	
O Beef	
O Chicken	
O Milk	
O Nuts	
O Rootcrops: ___________________________	
O Fruits: _______________________________	
Others:	

Part 6. Constraints

6.1 Can you tell me about the biggest problem you have about your livelihoods and what you do to try and mitigate them? *(If they talk about wild animals raiding their farms, take note of that, and then ask if there are still other problems.)*

____

____

____

____

____

(Ask the following questions, only if it is not yet 1 hour and 10 minutes since start of interview

6.2 Which place(s) in the landscape do you like because of its beauty? Please explain why.

Aisa O. Manlosa, Jan Hanspach, Jannik Schultner, Ine Dorresteijn, and Joern Fischer
Corresponding author: Aisa O. Manlosa, Faculty of Sustainability, Leuphana University Lüneburg, aisamanlosa@gmail.com

____

____

6.3 Which place(s) in the landscape do you use for recreation and relaxation? Please explain why.

____

____

That completes the survey. Thank you very much for sharing your time and this valuable information with us. Do you have any question for us before we close? Again, thank you and I wish you a good day.

(The interviewer should answer this after the interview.)

What is it about this household that is interesting and which I might want to come back for? And how do I find this household again?

____

____

Aisa O. Manlosa, Jan Hanspach, Jannik Schultner, Ine Dorresteijn, and Joern Fischer
Corresponding author: Aisa O. Manlosa, Faculty of Sustainability, Leuphana University Lüneburg, aisamanlosa@gmail.com
