# Supplementary material for: Livelihood strategies, capital assets, and food security in rural Southwest Ethiopia
Source: Food Secur. 2019 Jan 24;11(1):167–81. doi: 10.1007/s12571-018-00883-x (PMC6411135; doi:10.1007/s12571-018-00883-x)
Supplement: Supplementary file 3 — (PDF 85 kb) [file 12571_2018_883_MOESM3_ESM.pdf]

Online Resource 3 List of livelihood activities included in multivariate analyses and corresponding measurement in the production period preceding the survey.

Livelihood variables	Measurement
Barley	Yield (kg)
Coffee	Yield (kg)
Honey	Quantity collected (kg)
Home garden diversity	Number of plants in home gardens with important use
Khat	Presence-absence data
Legumes	Combined quantity of beans and peas (kg)
Milk	Quantity collected per day (liters)
Maize	Yield (kg)
Others	Combined presence-absence data on engagement in non-farm wage labor, farm wage labor, receiving remittance, engagement in petty trade, and selling of livestock
Sorghum	Yield (kg)
Teff	Yield (kg)
Wheat	Yield (kg)

Aisa O. Manlosa, Jan Hanspach, Jannik Schultner, Ine Dorresteijn, and Joern Fischer
Corresponding author: Aisa O. Manlosa, Faculty of Sustainability, Leuphana University Lüneburg, aisamanlosa@gmail.com
