# Supplementary material for: Livelihood strategies, capital assets, and food security in rural Southwest Ethiopia
Source: Food Secur. 2019 Jan 24;11(1):167–81. doi: 10.1007/s12571-018-00883-x (PMC6411135; doi:10.1007/s12571-018-00883-x)
Supplement: Supplementary file 4 — (PDF 156 kb) [file 12571_2018_883_MOESM4_ESM.pdf]

Online Resource 4 Mathematical formulas for the generalized linear model and log transformation of livelihood variables

A generalized linear model was used to test the effect livelihood strategy, household characteristics, *kebele*, and survey data on the dependent variable HFIAS score (food security measure). The formula is as follow:

$\log(hfiasscore_i) = \beta_0 + \beta_1 x_{1i} + \beta_2 x_{2i} + \beta_3 x_{3i} + \beta_4 x_{4i} + \beta_5 x_{5i} + \beta_6 x_{6i} + \beta_7 x_{7i} + \beta_8 x_{8i} + \beta_9 x_{9i} + \beta_{10} x_{10i} + \beta_{11} x_{11i} + \beta_{12} x_{12i} + \beta_{13} x_{13i} + \beta_{14} x_{14i} + \beta_{15} x_{15i} + \beta_{16} x_{16i} + \beta_{17} x_{17i}$

with x_1=1 if livelihood strategy type is 2 (otherwise 0), x_2=1 if livelihood strategy type is 3 (otherwise 0), x_3=1 if livelihood strategy type is 4 (otherwise 0), x_4=1 if livelihood strategy type is 5 (otherwise 0), x_5=1 if sex=male (otherwise 0), x_6=date, x_7=age, x_8=1 if education cluster is 1 (otherwise 0), x_9=1 if education cluster is 2 (otherwise 0), x_{10}=1 if education cluster is 3 (otherwise 0), x_{11}=household size, x_{12}=number of ill household members, x_{13}=1 if kebele=GBW (otherwise 0), x_{14}=1 if kebele=GQH (otherwise 0), x_{15}=1 if kebele=GQQ (otherwise 0), x_{16}=1 if kebele=SDM (otherwise 0), x_{17}=1 if kebele=SGB (otherwise 0)

The variables used for the multivariate analysis were log-transformed to meet requirements of normality. The formula is shown below:

$y_{ij} = w_{1i}*gardendiversity_j + w_{2i}*milk_j + w_{3i}*honey_j + w_{4i}*\log(maizeyield_j+1) + w_{5i}*\log(teffyield_j+1) + w_{6i}*\log(sorghumyield_j+1) + w_{7i}*\log(coffeeyield_j+1) + w_{8i}*\log(wheatyield_j+1) + w_{9i}*\log(barleyyield_j+1) + w_{10i}*khat_j + w_{11i}*otherincome_j + w_{12i}*\log(legumes_j+1)$

With w denoting weights and for i=1,…,12 principal components and j=1,….,337 households

Aisa O. Manlosa, Jan Hanspach, Jannik Schultner, Ine Dorresteijn, and Joern Fischer
Corresponding author: Aisa O. Manlosa, Faculty of Sustainability, Leuphana University Lüneburg, aisamanlosa@gmail.com
