# Supplementary material for: Livelihood strategies, capital assets, and food security in rural Southwest Ethiopia
Source: Food Secur. 2019 Jan 24;11(1):167–81. doi: 10.1007/s12571-018-00883-x (PMC6411135; doi:10.1007/s12571-018-00883-x)
Supplement: Supplementary file 5 — (PDF 155 kb) [file 12571_2018_883_MOESM5_ESM.pdf]

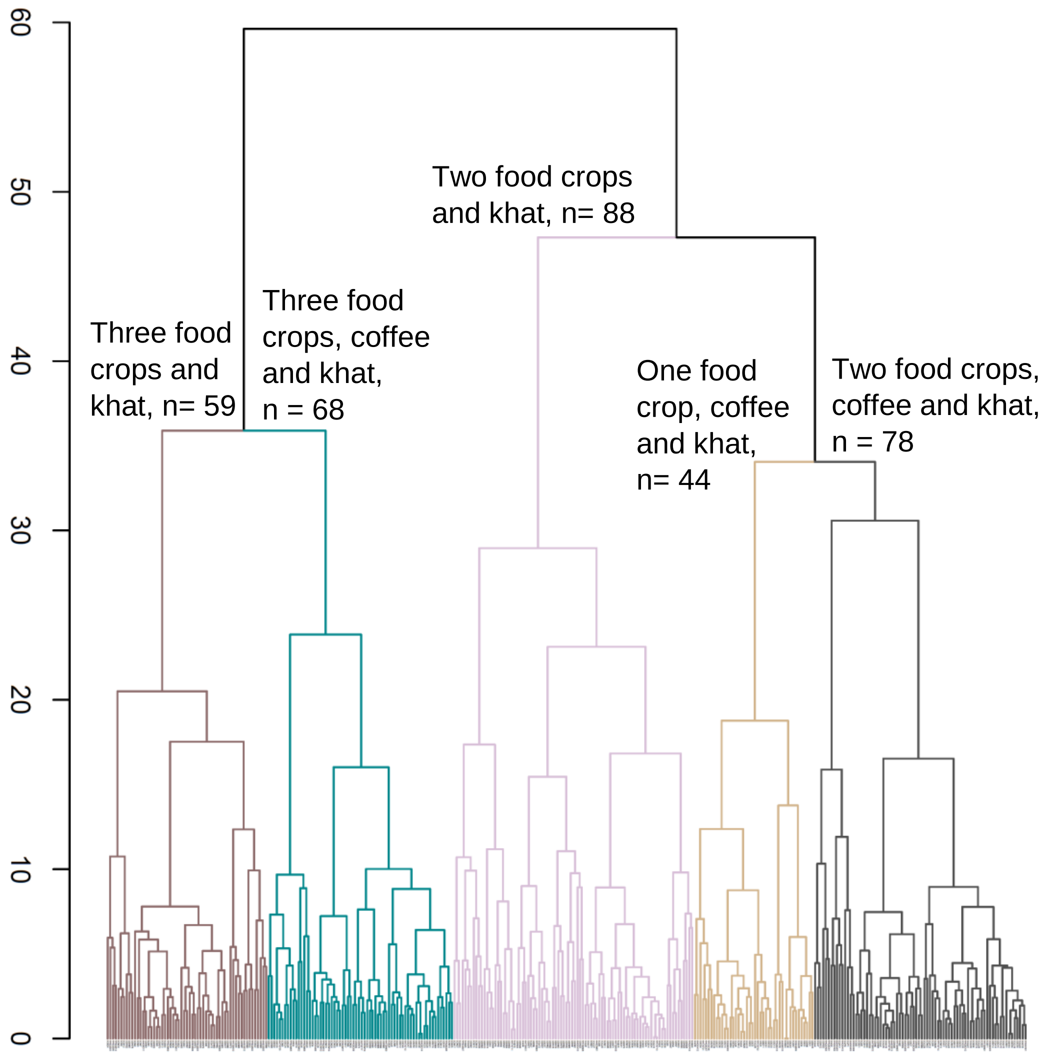


**Online Resource 5** Full dendogram of livelihood strategies from cluster analysis. For the livelihood activities that compose each cluster or livelihood strategy, see Figure 1.

Livelihood strategies, capital assets, and food security in rural southwest Ethiopia
Food Security
Aisa O. Manlosa, Jan Hanspach, Jannik Schultner, Ine Dorresteijn, and Joern Fischer
Corresponding author: Aisa O. Manlosa, Faculty of Sustainability, Leuphana University Lüneburg, aisamanlosa@gmail.com
