# Supplementary material for: Livelihood strategies, capital assets, and food security in rural Southwest Ethiopia
Source: Food Secur. 2019 Jan 24;11(1):167–81. doi: 10.1007/s12571-018-00883-x (PMC6411135; doi:10.1007/s12571-018-00883-x)
Supplement: Supplementary file 6 — (PDF 258 kb) [file 12571_2018_883_MOESM6_ESM.pdf]

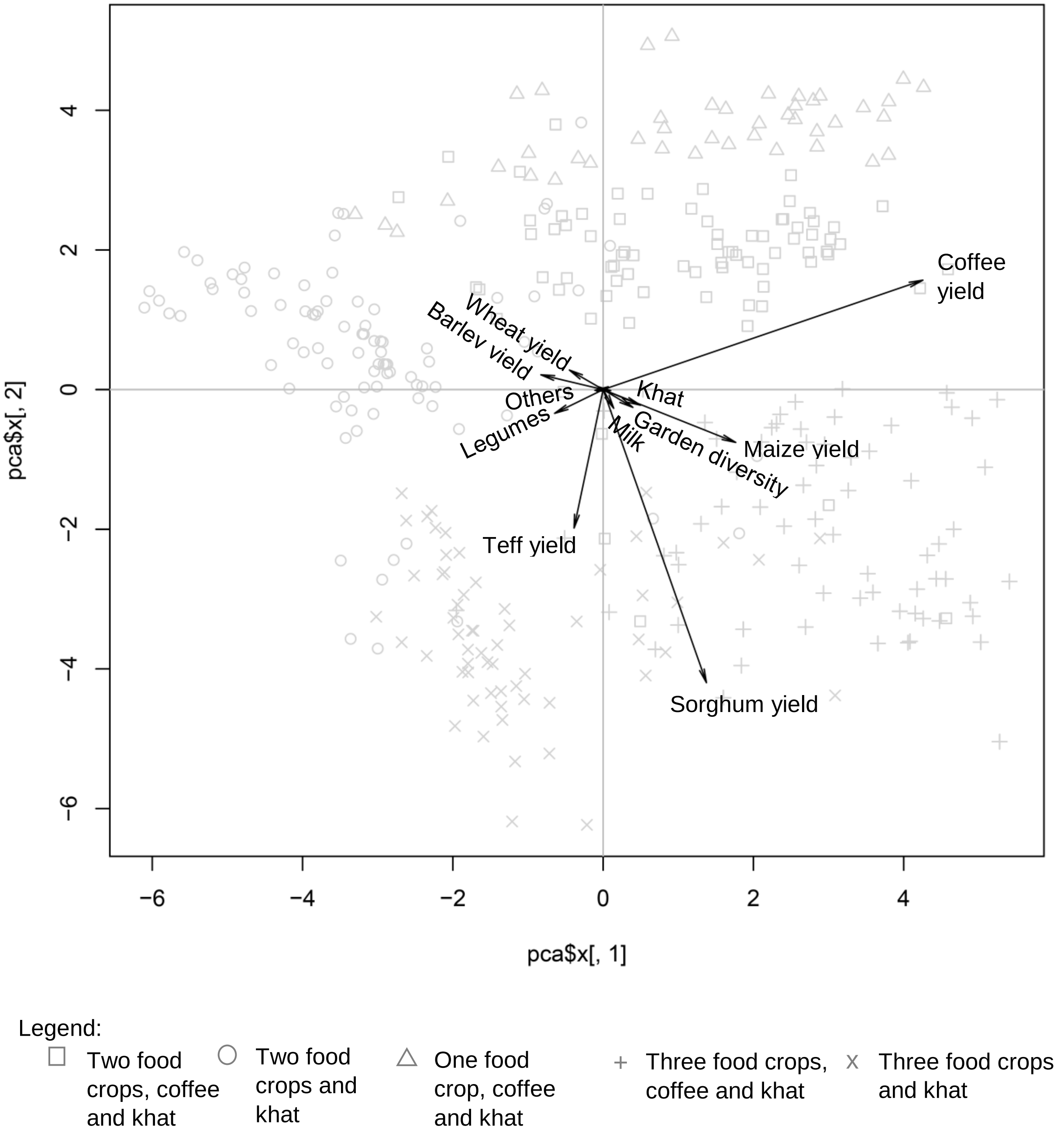

Online Resource 6 PCA plot of livelihood activities. The symbols indicate the livelihood strategies that households belong to. The first and second axis of the PCA accounted for 26% and 23% of variation in the data, respectively. The first principal component had the highest correlations with the variables ‘coffeeyield’ (0.85), ‘maizeyield’ (0.35), and ‘sorghumyield’ (0.27). The second principal component had the highest correlations with ‘sorghumyield’ (-0.84), ‘teffyield’ (-0.40) and ‘coffeeyield’ (0.31).

Livelihood strategies, capital assets, and food security in rural southwest Ethiopia
Food Security
Aisa O. Manlosa, Jan Hanspach, Jannik Schultner, Ine Dorresteijn, and Joern Fischer
Corresponding author: Aisa O. Manlosa, Faculty of Sustainability, Leuphana University Lüneburg, aisamanlosa@gmail.com
