# Supplementary material for: Livelihood strategies, capital assets, and food security in rural Southwest Ethiopia
Source: Food Secur. 2019 Jan 24;11(1):167–81. doi: 10.1007/s12571-018-00883-x (PMC6411135; doi:10.1007/s12571-018-00883-x)
Supplement: Supplementary file 7 — (PDF 166 kb) [file 12571_2018_883_MOESM7_ESM.pdf]

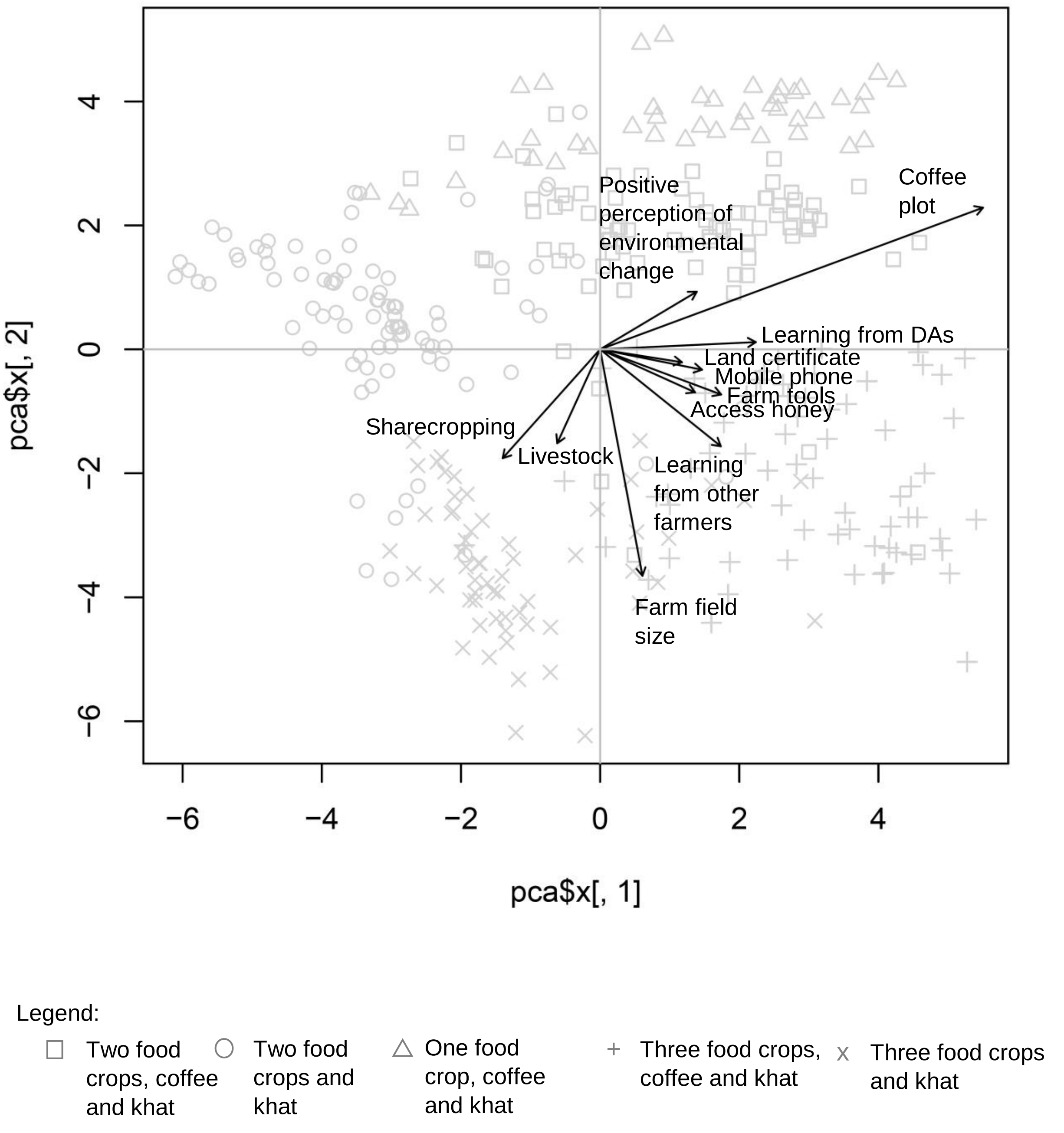

Online Resource 7 The full range of capital asset variables that correlated significantly with the PCA axes at p<0.01. Different capital assets have a strong association with certain types of livelihood strategies. For example, livelihood strategies with more food crops are linked with having a bigger farm field relative to others in the area, and having access to more types of capital assets.

Livelihood strategies, capital assets, and food security in rural southwest Ethiopia
Food Security
Aisa O. Manlosa, Jan Hanspach, Jannik Schultner, Ine Dorresteijn, and Joern Fischer
Corresponding author: Aisa O. Manlosa, Faculty of Sustainability, Leuphana University Lüneburg, aisamanlosa@gmail.com
