# Supplementary material for: Livelihood strategies, capital assets, and food security in rural Southwest Ethiopia
Source: Food Secur. 2019 Jan 24;11(1):167–81. doi: 10.1007/s12571-018-00883-x (PMC6411135; doi:10.1007/s12571-018-00883-x)
Supplement: Supplementary file 8 — (PDF 261 kb) [file 12571_2018_883_MOESM8_ESM.pdf]

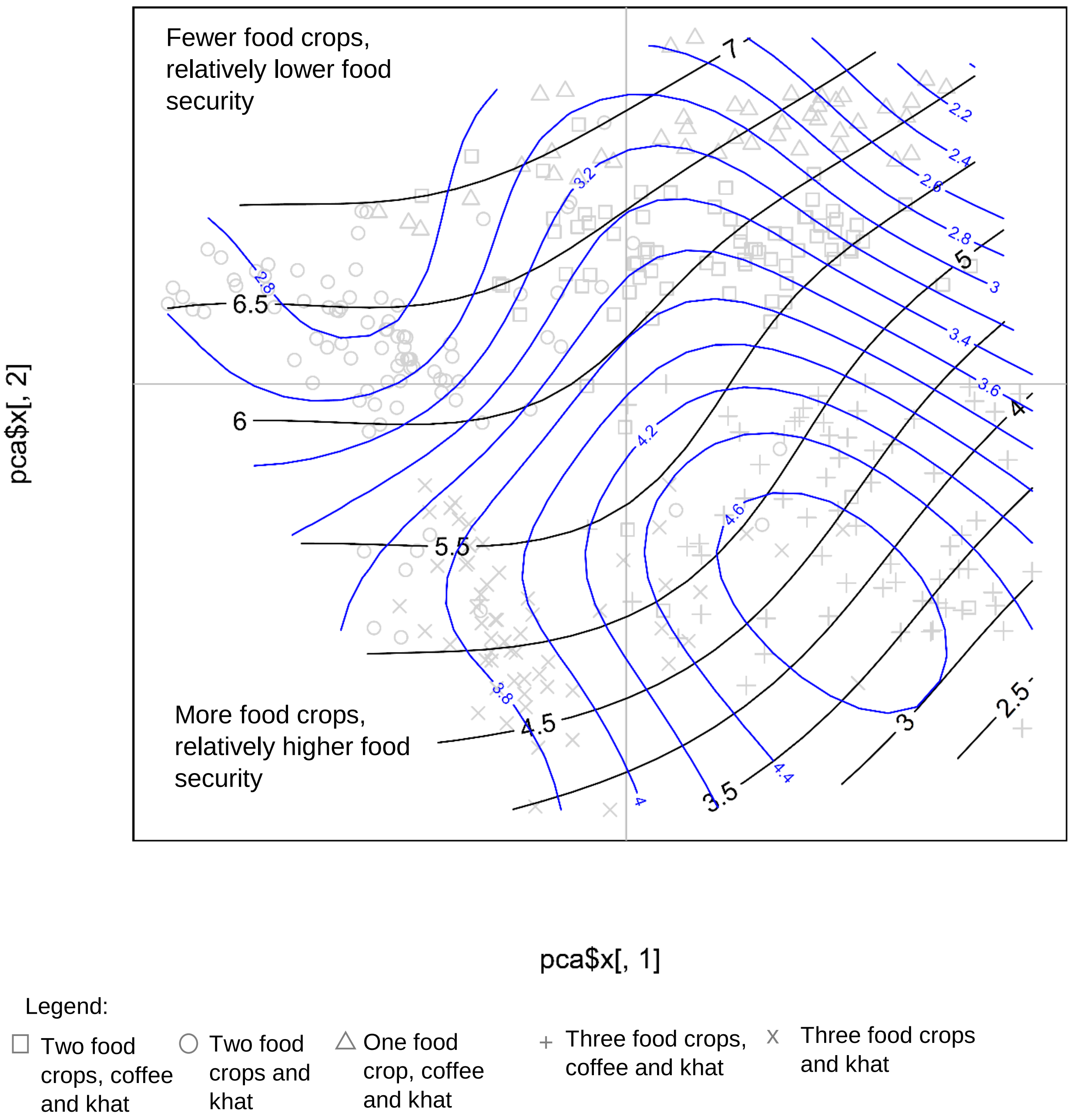

Online Resource 8 Visualization of the gradient of food security as measured by HFIAS scores and number of crops in specific livelihood strategies. The black contour lines are the HFIAS scores. High HFIAS scores indicate low food security and low HFIAs scores indicate high food security. The blue contour lines indicate the number of crops. Households with higher number of crops have higher food security.

Aisa O. Manlosa, Jan Hanspach, Jannik Schultner, Ine Dorresteijn, and Joern Fischer
Corresponding author: Aisa O. Manlosa, Faculty of Sustainability, Leuphana University Lüneburg, aisamanlosa@gmail.com
